# Reply to letter to editor by McCullough

**DOI:** 10.1080/07853890.2021.1878273

**Published:** 2021-01-29

**Authors:** Amandeep Goyal, Pankaj Bansal

**Affiliations:** a University of Kansas Medical Center, Kansas City, KS, USA; b Mayo Clinic Health System, Eau Claire, WI, USA

We reviewed the concerns raised by Dr. McCullough about our article, “Hydroxychloroquine: A comprehensive review and its controversial role in coronavirus disease 2019”, and appreciate this opportunity to clarify these concerns for Dr. McCullough and other readers [1].

Dr. McCullough has commented about “a large white scar in the short-axis image of the left ventricle similar to that of a myocardial infarction” in figure 4 of the article. We would like to mention that this is an inaccurate interpretation of the figure and article. The image key for this image clearly mentions that the white discolouration is a depiction of “lysosomal storage” which causes cardiomyopathy and not a “scar”. We agree with Dr. McCullough that cardiac scarring or any changes in the myocardium similar to that of myocardial infarction is not an adverse effect of hydroxychloroquine and has not been suggested by us in the article anywhere. Hydroxychloroquine induced cardiomyopathy is a well-known phenomenon and the article very well depicts the pathophysiological basis of cardiomyopathy caused by hydroxychloroquine. Dr. McCullough has mentioned that our graphic depiction of HCQ and myocardial scar in the figure is misleading to the readership and frightening to patients. We would like to differ, and our novel images clarify the pathophysiological mechanism of cardiomyopathy which can be caused by hydroxychloroquine. The detailed pathophysiology has been well described in the manuscript itself in addition to the graphic depiction. The intention of the paper is not to mislead anyone or frighten patients, but to generate a thorough understanding of the pathophysiological basis of various toxicities caused by this medication, as well as their risk factors. A thorough reading of the entire article in addition to the graphics can clarify any such doubts for the readers.

Further, Dr. McCullough has raised concerns that the original article suggests that cardiomyopathy is caused by hydroxychloroquine within 5–30 days. We would like to clarify that the original article clearly mentions that it takes many years for hydroxychloroquine to cause cardiomyopathy, which is a very rare event. Further, risk factors for the development of hydroxychloroquine-induced cardiomyopathy were described by us as well. We agree with Dr. McCullough that hydroxychloroquine-induced cardiomyopathy does not occur within 30 days of the use of hydroxychloroquine and would again like to stress that this is clearly mentioned and clarified in the article. Dr. McCullough has also raised concerns about a reference by Stein suggesting we referenced this citation in support of cardiomyopathy [2]. We would like to clarify that this exact reference by Stein as mentioned by the author is actually reference number 54 in the article, cited under “other adverse effects” explaining the neuromyotoxicity of hydroxychloroquine and not in the section for cardiomyopathy. This reference has not been used in the article to support any statement about cardiomyopathy. To support the evidence behind cardiomyopathy caused by hydroxychloroquine, references from numbers 36 to 48 cited in the original article can be reviewed [1].

Finally, Dr. McCullough mentions that “hydroxychloroquine (HCQ), which at the moment is the most widely used prophylactic and therapeutic agent for SARS-CoV-2 infection (COVID-19) in the world”. However, this statement lacks enough supporting evidence, and the references provided by Dr. McCullough to support this statement are weak. None of the recently performed randomised controlled clinical trials have shown any efficacy of hydroxychloroquine for treatment or prophylaxis of COVID-19 [3–6]. Several health agencies including the Centres for Disease Control, Food and Drug Administration, European Medical Agency, American College of Physicians, Infectious Disease Society of America, and National Institutes of Health have already recommended against the use hydroxychloroquine for COVID-19 outside clinical trials [7–11].
